# The Silencing of Pokemon Attenuates the Proliferation of Hepatocellular Carcinoma Cells *In Vitro* and *In Vivo* by Inhibiting the PI3K/Akt Pathway

**DOI:** 10.1371/journal.pone.0051916

**Published:** 2012-12-26

**Authors:** Chan-Chan Lin, Jing-Ping Zhou, Yun-Peng Liu, Jing-Jing Liu, Xiao-Ning Yang, Amarsanaa Jazag, Zhi-Ping Zhang, Bayasi Guleng, Jian-Lin Ren

**Affiliations:** 1 Department of Gastroenterology, Zhongshan Hospital, Xiamen University, Xiamen, China; 2 Faculty of Clinical Medicine, Medical College of Xiamen University, Xiamen, China; 3 National Institute of Medical Research, 3rd General Hospital, Ulaanbaatar, Mongolia; Institute of Hepatology London, United Kingdom

## Abstract

Pokemon (POK erythroid myeloid ontogenic factor), which belongs to the POK protein family, is also called LRF, OCZF and FBI-1. As a transcriptional repressor, Pokemon assumes a critical function in cellular differentiation and oncogenesis. Our study identified an oncogenic role for Pokemon in human hepatocellular carcinoma (HCC). We successfully established human HepG2 and Huh-7 cell lines in which Pokemon was stably knocked down. We demonstrated that Pokemon silencing inhibited cell proliferation and migration. Pokemon knockdown inhibited the PI3K/Akt and c-Raf/MEK/ERK pathways and modulated the expression of various cell cycle regulators in HepG2 and Huh-7 cells. Therefore, Pokemon may also be involved in cell cycle progression in these cells. We confirmed that Pokemon silencing suppresses hepatocellular carcinoma growth in tumor xenograft mice. These results suggest that Pokemon promotes cell proliferation and migration in hepatocellular carcinoma and accelerates tumor development in an Akt- and ERK-signaling-dependent manner.

## Introduction

Hepatocellular carcinoma (HCC) is one of the most prevalent cancers worldwide, and the disease has a poor prognosis. The molecular mechanisms of hepatocarcinogenesis involve various oncogenes, tumor-suppressor genes and growth factor genes [1]. In HCC, mutations in p53, microdeletions of p14ARF and increases in Mdm2 expression occur at different frequencies. In addition, the CDKIs p15^INK4b^, p16^INK4a^ and p21^Cip1^ are often inactivated in this cancer [2].

Pokemon (POK erythroid myeloid ontogenic factor), which is encoded by the ZBTB7A gene, has recently been identified as a POK transcription factor with proto-oncogenic activity. Pokemon contains a POZ domain at the N terminus and a Krüppel-type (C2H2) zinc finger domain at the C terminus [3]–[4]. Pokemon is overexpressed in non-small cell lung carcinoma and malignant gliomas, and has been observed to be expressed in human breast carcinomas; the nuclei of human colon, renal, and thymoma tumor cells; and hepatocellular carcinomas [5]. Nevertheless, few studies have assessed the role of Pokemon in HCC [6].

A high level of Pokemon expression suppresses the transcription of the tumor suppressor protein p14ARF. Mdm2 is reactivated to alleviate p14ARF suppression, which reduces p53 expression and leads to tumorigenesis [7]. The POZ domain of Pokemon interacts with the RHD (Rel homology domain) of the p65 subunit of nuclear factor-κB (NF-κB) to enhance NF-κB-mediated transcription [8]. Recently, the impact of Pokemon on the cell cycle has been investigated. Through its POZ domain, Pokemon represses the transcription of the Rb gene, which is implicated in cell cycle arrest. The POZ domain recruits co-repressor-histones and exhibits binding competition with Sp1 at the Rb gene promoter [9]. Pokemon transcriptionally represses p21, which is a key regulator of mammalian cell cycle arrest [10]. Pokemon activity is mediated by direct binding competition with the Sp1/3 GC-box and the p53-responsive elements of p21 [11].

Previous studies have demonstrated that the PI3K/Akt pathway is involved in the pathogenesis of HCC [12]–[13]. In addition, the MEK/ERK pathway enhances proliferation and inhibits apoptosis in HepG2 cells [14] and promotes the development of hepatocellular carcinoma in vivo [15]. Moreover, PTEN, a tumor suppressor gene, is frequently mutated or deleted in various human cancers, including liver cancer. PTEN mainly localizes to the cytoplasm and negatively regulates PI3K/AKT signaling. PTEN also localizes to the nucleus, where it regulate the protein level and transcriptional activity of p53 [16]. Recent studies have focused on the synergy between the PI3K/Akt and MEK/ERK pathways in HCC [17], [18]. However, the mechanism underlying these synergistic actions remains unknown. In this study, we determined how Pokemon participates in the development of hepatocellular carcinoma by regulating the PI3K/Akt and MEK/ERK pathways in HCC cells.

## Materials and Methods

### Ethics statement

This study was approved by the Ethics Committee (No: 20081009) of Zhongshan Hospital, affiliated with Xiamen University in Xiamen, Fujian Province, China. Written consent was obtained from all participants who were involved in the study. All procedures involving experimental animals were performed in accordance with protocols that were approved by the Committee for Animal Research of Xiamen University and complied with the Guide for the Care and Use of Laboratory Animals (NIH publication No. 86-23, revised 1985).

### Immunohistochemistry

We collected 20 paraffin-embedded HCC and 20 tumor-adjacent noncancerous tissue specimens as controls from the Department of Pathology of Zhongshan Hospital at Xiamen University in Xiamen, China. All of the specimens were validated by pathological diagnosis. The tumors were classified according to the WHO classification system and staged using the pTNM system. Five-micron-thick paraffin sections were either stained with hematoxylin and eosin (H&E) or analyzed for Pokemon expression by immunohistochemistry, which was performed according to the manufacturer's instructions. The primary anti-Pokemon (1∶300 dilution) antibody was purchased from AB Biotec, USA. The reactions were visualized using diaminobenzidine as a chromogenic substrate. The sections were counterstained using hematoxylin, then cleared and mounted. The staining score was calculated based on the percent positive area (no positive staining = 0; less than 25% = 1 point; 25–50% = 2 points; 51–75% = 3 points; and more than 75% = 4 points) multiplied by the staining intensity (weak = 1; moderate = 2; strong = 3 very strong = 4). Five fields of view were randomly selected for each sample, and the average positive points for the cases are the actual positive points. Samples with less than 2 points were classified as negative, and samples with 2 or more points were classified as positive.

### Cell lines and reagents

The human hepatocellular carcinoma (HCC) cell line HepG2, Huh-7 and hepatocyte HL-7702 cells were grown in DMEM (Invitrogen) supplemented with 10% fetal bovine serum (GIBCO) and 100 IU/MI penicillin-streptomycin. All cell lines were purchased from ATCC (American Type Culture Collection, United States).

### RNA extraction and semi-quantitative RT-PCR

Total RNA was extracted from cells using TRIzol reagent (Invitrogen, United States). RNA extraction was performed according to the manufacturer's instructions. The RNA samples were dissolved in nuclease-free water, and the concentration was measured by detecting the absorbance at 260 and 280 nm. The reverse transcription (RT) reaction for first-strand cDNA synthesis was performed using reverse transcriptase (Bio-Rad) with 2 µg of total RNA. The primers for the Pokemon gene (synthesized by Invitrogen) were as follows: forward primer, 5′-GGGGACAGCGACGAGGAG-3′; reverse primer, 5′-CGTAGTTGTGGGCAAAGG-3′. The following primers for β-actin were also used: forward primer, 5′-CTCCATCCTGGCCTCGCTGT-3′; reverse primer, 5′-GCTGTCACCTTCACCGTTCC-3′. RT-PCR experiments were performed in 25 µl volumes of PCR buffer (Fermentas) with an initial denaturation at 95°C for 5 min followed by 30 cycles at 94°C for 40 s, 57.7°C for 40 s, and 72°C for 40 s, with a final extension at 72°C for 10 min.

### Protein extraction and Western blot analysis

Total protein was extracted from cells and tissue specimens using the Mammalian Cell Lysis Reagent (Fermentas) according to the manufacturer's protocol. The proteins were resolved using 10%, 12% or 15% sodium dodecyl sulfate polyacrylamide gel electrophoresis and analyzed using the appropriate antibodies. Antibodies against Akt, p-Akt(473), ERK, p-ERK, p-ERK1/2 (Thr202/Tyr204), PTEN, p-PTEN, c-Raf, CDK4, CDK6, Cyclin D1, Cyclin D3, p15, p16, p21, GSK-3β (Ser9), P-GSK-3β (Ser9), and tubulin were purchased from Cell Signaling Technologies (United States). Antibodies against Pokemon, β-actin, and GAPDH were purchased from Abcam (United Kingdom).

### The establishment of stable Pokemon knockdown cell lines

A plasmid encoding a short interfering RNA (siRNA) targeting Pokemon was constructed as previously described [19]. Stable knockdown cells were also established as previously described [20]. Briefly, the two pairs of siRNA sequences (siPokemon-1 sequences: forward primer, 5′-CACCGCTAGGGGAAGTACT TTAAACGTGTGCTGTCCGTTTAAAGTGCTTCTCCTGGCTTTTT-3′; reverse primer, 5′-GCATAAA-AAGCCAGGAGAAGCACTTTAAACGGACAGCACACGTTTAAAGTACTTCCCCTAGC-3′ and siPokemon-2 sequences: forward primer, 5′-CACCAGTAGAATGTGTACGGGATAC-GTGTGCTGTCCGTATCTCGTCACGTTCTGCTTTTTT-3′; reverse primer, 5′-GCATAAAAAG-CAGAACGTGTACGAGATACGGACAGCACACGTATCCCGTACA-CATTCTACT-3′) for the Pokemon gene were selected using an algorithm as described in our previous study.

Both HepG2 and Huh-7 cells were transfected with siPokemon (pcPUR+U6-siPokemon) or a PU6 (pcPUR+U6-siRenilla) plasmid using the Lipofectamine 2000 transfection reagent. Puromycin (9.0 µg/ml) was used to screen stably transfected clones. The expression of the Pokemon protein was examined by Western blotting analysis using an antibody against Pokemon to validate the efficiency of the constructs to inhibit target gene expression; these experiments were repeated three times. The cell lines that were stably transfected and exhibited effective downregulation of the Pokemon gene were named HepG2-siPokemon and Huh-7-siPokemon, and the cell lines that were stably transfected with the control plasmid were named HepG2-Pu6 and Huh-7-Pu6.

### Cell proliferation assay

The methyl thiazolyl tetrazolium (MTT) (Sigma, St. Louis, MO, USA) colorimetric assay was used to screen for cell proliferation. Briefly, cells were seeded in four 96-well plates at a density of 2×10^3^ cells/well. One plate was taken out at the same time every day after the cells had adhered to the well. A 20 µl aliquot of MTT (5 mg/mL) was added to each well, and the cells were incubated under the culture conditions for a further 4 h. The medium was removed, and the formazan precipitate was solubilized in 150 mL dimethylsulfoxide (DMSO). The absorbance at a wavelength of 490 nm was measured using a microplate reader. A BrdU assay was performed according to the manufacturer's instructions (Cell Signaling Technology). Cells were seeded at 1×10^4^ cells/well in a 96-well plate and incubated overnight. The cells were then incubated for 24 hours. Finally, 10 µM BrdU was added to the plate, and the cells were incubated for 4 hours. The absorbance at a wavelength of 450 nm was measured using a microplate reader. All experiments were performed in triplicate.

### Cell migration and wound healing assay

The cell migration assay was performed in a Boyden chamber, which consists of a cylindrical cell culture insert nested inside the well of a cell culture plate. The insert contains a polycarbonate membrane at the bottom with a defined pore size. We seeded HepG2-siPokemon cells, Huh-7-siPokemon cells or control cells in the top of the insert at a density of 5×10^4^ cells/ml in 200 µl of serum-free DMEM medium, while a total of 600 µl of DMEM containing 5% fetal bovine serum was placed in the well below. Each sample was plated into three duplicate wells. Migratory cells move through the pores toward the serum below and can be stained with Basic Violet. The cells on the upper surface of the filter were removed with a cotton swab. The migrated cells on the lower surface of the filter were rinsed with 33% acetic acid. The numbers of migrated cells were counted and quantified under a microscope. A wound-healing assay was performed according to a previously published protocol [21].

### FCM analysis of the cell cycle

To analyze the cell cycle profiles of each cell line, HepG2-siPokemon, Huh-7-siPokemon and control cells were labeled with a propidium iodide (100 µg/ml, Sigma) solution containing RNase A (100 µg/ml) according to the manufacturer's instructions (KeyGEN Biotech). The samples were subjected to FACS analysis, and the data were analyzed using ModFit LT v.2.0.

### Xenograft experiment

To determine the oncogenicity of Pokemon, 16 approximately 4- to 6-month-old male nude mice were randomly divided into two groups. The nude mice were subcutaneously inoculated with 6×10^6^ HepG2-siPokemon or HepG2-Pu6 cells per mouse in the gluteal region. The tumor size was assessed every four days using calipers, and the measured values were used to calculate the tumor volume according to the formula [length (mm)×width (mm)^2^]/2 [20]. After seventeen days, the mice were euthanized to measure tumor weights.

### Statistical analyses

Statistical analyses were performed using SPSS (Statistical Package for the Social Sciences) v13.0 (SPSS, Inc.) and GraphPad Prism 5. The independent-samples *t*-test and paired-samples *t*-test were used to compare the data. All of the values were expressed as the mean ± SD. Differences were considered to be statistically significant at *P*<0.05.

## Results

### Pokemon protein expression is upregulated in HCC tissues

**Figure 1:**
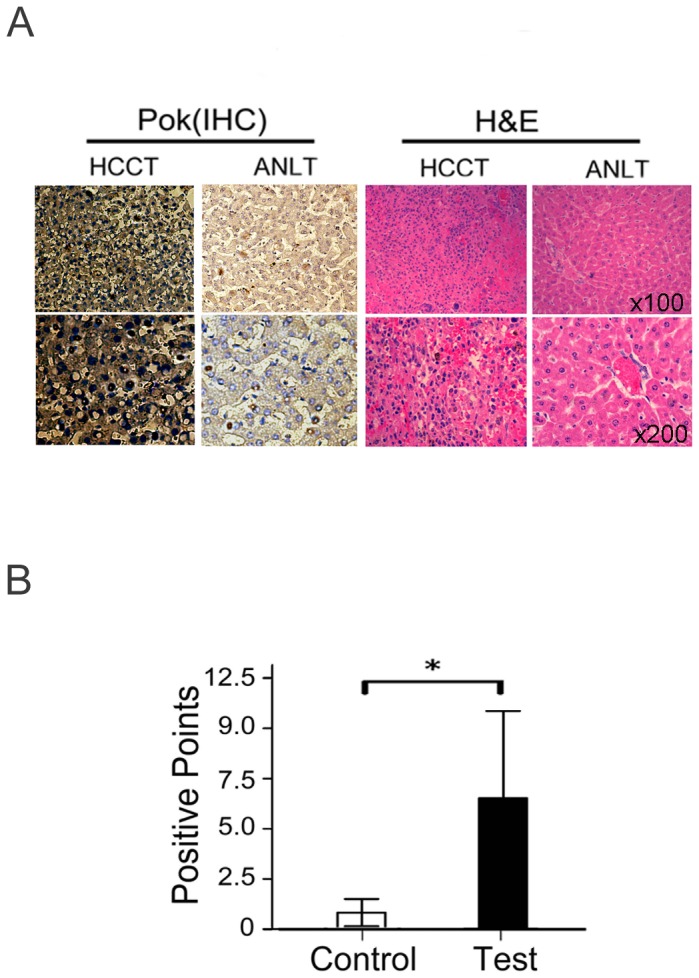
Immunohistochemical analysis of Pokemon expression in HCC tissue (Test Group) and adjacent noncancerous liver tissue (Control Group). (**A**). Typical representative immunohistochemical results from one pair of HCC tissue (HCCT) and adjacent noncancerous liver tissue (ANLT) using an anti-Pokemon antibody. Pokemon was stained brown in granules. (**B**). A bar graph representing the relative expression level of Pokemon in the HCCT and ANLT groups, as evaluated by positive staining points. (A paired-samples *t*-test was used to compare the data from the two groups, *P<0.01).

Immunohistochemical analysis showed that Pokemon was expressed diffusely in the cytoplasm and was rarely present in the nucleus (Fig. 1A). Pokemon was markedly expressed in 16/20 (80.0%) of HCC tissues, and the expression levels were higher in HCC than in adjacent noncancerous liver tissues (*P*<0.01, Table 1). The relative expression levels of Pokemon in the HCC tissue group (Test group) and the corresponding pathologically noncancerous liver tissue group (Control group) were evaluated based on positive staining points. We observed a statistically significant increase in Pokemon expression in the test group compared with the control group (Fig. 1B, *P*<0.01). These data suggest that the Pokemon gene may play an important role in HCC development.

**Table 1:** The expression of Pokemon protein in HCC.

Group	Cases (n)	Positive (n)	Negative (n)	Positive rate	Positive point	*P*
Test	20	16	4	80.00%	7.4±4.838	<0.01
control	20	5	15	25.00%	0.95±0.759	

Paired-Sample T Test was used to statistic.

### Human HepG2 and Huh-7 cells expressed high levels of Pokemon, which were successfully knocked down by siRNA

**Figure 2:**
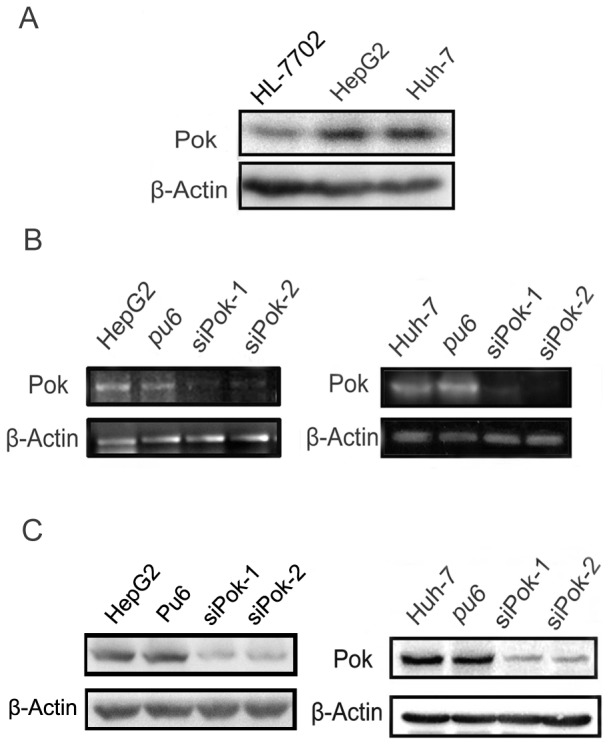
Pokemon protein expressions in human HepG2 and Huh-7 cell lines and the siRNA silencing effects of Pokemon. (**A**) Western blot analysis of Pokemon protein expression in the HepG2 and Huh-7 cell lines. The Pokemon expression in HL-7702 cells was used as a control, and the expression of β-actin was used as an internal control; (**B**) RT–PCR analysis of the Pokemon RNA level in HepG2 and Huh-7 cells that were stably transfected with Pokemon siRNA or the Pu6 vector control. The Pokemon expression levels in wild type HepG2 and Huh-7 cells were used as positive controls. (**C**) Western blot analysis to confirm the siRNA silencing of Pokemon, with β-actin used as a loading control.

We used Western blot analysis to determine the expression of Pokemon (labeled as [Pok] in the figure) protein in wild-type human HepG2 and Huh-7 cells and in HL-7702 cells, which were used as a control (Fig. 2A). We transfected HepG2 and Huh-7 cells with Pokemon siRNA and the Pu6 empty vector (control) to stably establish HepG2-siPokemon-1/2 and Huh-7-siPokemon-1/2 cells and control HepG2-Pu6 and Huh-7-Pu6-cells. We used PCR and Western blot analyses to verify Pokemon downregulation at the RNA and protein levels, respectively. Our results show that Pokemon RNA and protein expression were downregulated in the HepG2-siPokemon and Huh-7-siPokemon cells compared with the HepG2-pu6 and Huh-7-Pu6 cells (Fig. 2B, C). Because the downregulation was stronger in the siPokemon-2 cells, we employed HepG2-siPokemon-2 and Huh-7-siPokemon-2 cells to perform the following studies. These cells are labeled as “siPok” and “Pu6” cells in the figure.

### The knockdown of endogenous Pokemon attenuates cell proliferation and migration in hepatocellular carcinoma cells

**Figure 3:**
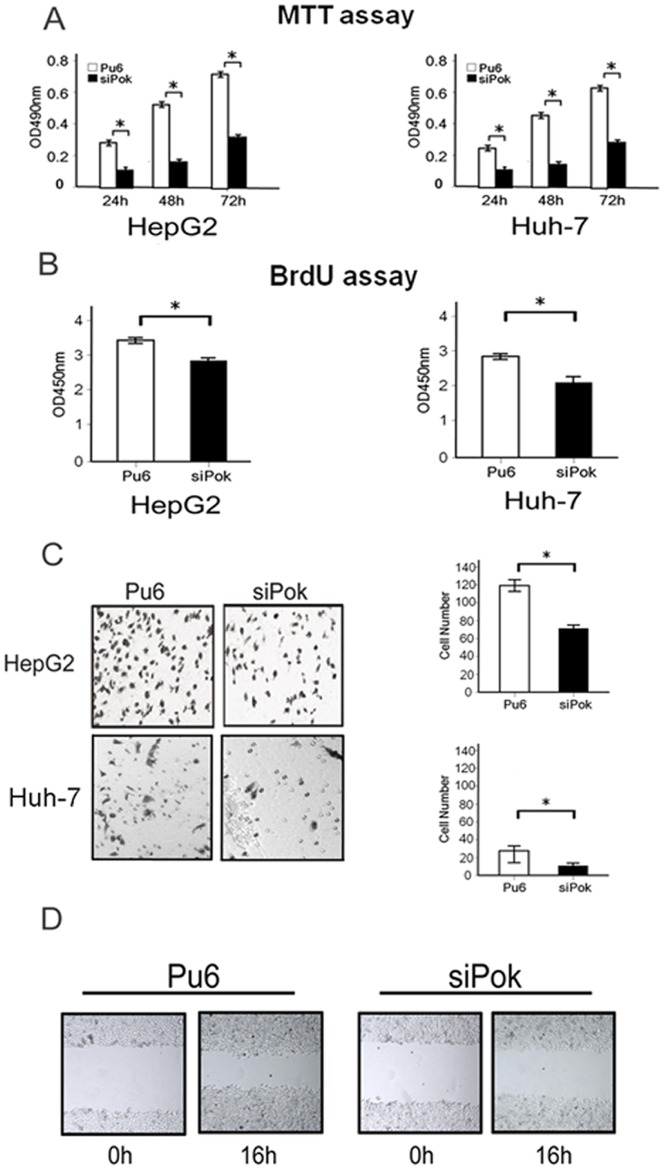
Functional changes in HepG2 and Huh-7 cells induced by Pokemon silencing. (**A**). MTT assay analysis of cell proliferation in HepG2-siPokemon, Huh-7-siPokemon and control cells (*P<0.01). (**B**). BrdU assay analysis of cell proliferation in HepG2-siPokemon, Huh-7-siPokemon and control cells after 24 hours of incubation (*P<0.01). (**C**). Representative photographs from the migration assay and a histogram of the quantification of HepG2-siPokemon, Huh-7-siPokemon and control cells (*P<0.01). (**D**). Representative photographs from the wound healing assay in HepG2-siPokemon and control cells.

Because the role of Pokemon in the development of hepatocellular carcinoma has rarely been examined, we investigated the effect of Pokemon on the proliferation of HepG2 and Huh-7 cells. MTT assays were performed on HepG2-siPokemon cells, Huh-7-siPokemon cells and control cells. The results showed a reduction in the proliferation rate of the HepG2-siPokemon and Huh-7-siPokemon cells compared with the control cells at 24 h, 48 h and 72 h (Fig. 3A). We evaluated the proliferative capacity of the HepG2-siPokemon and Huh-7-siPokemon cells using a BrdU cell proliferation assay. The BrdU staining after incubation for 24 hours was consistent with the MTT assay results and demonstrated that the silencing of Pokemon inhibits the proliferation of HepG2 and Huh-7 cells compared with control cells (Fig. 3B).

A transwell migration assay was used to investigate the effect of Pokemon on cell migration. Both HepG2-siPokemon and Huh-7-siPokemon cells displayed significantly decreased migration compared with control cells (Fig. 3C). We also performed a wound healing assay in HepG2-siPokemon cells; as expected, Pokemon silencing inhibited this process (Fig. 3D). In addition, we analyzed the cell cycle profile of HepG2-siPokemon and Huh-7-siPokemon cells using FCM analysis, but no significant changes were observed between siPokemon and control cells (data not shown). Taken together, these results demonstrate that Pokemon is essential for HepG2 cell proliferation and migration.

### Pokemon regulates the phosphatidylinositol 3-kinase (PI3K)/Akt and Raf/MEK/ERK pathways, and its downstream effectors are also involved in the regulation of cell proliferation

**Figure 4:**
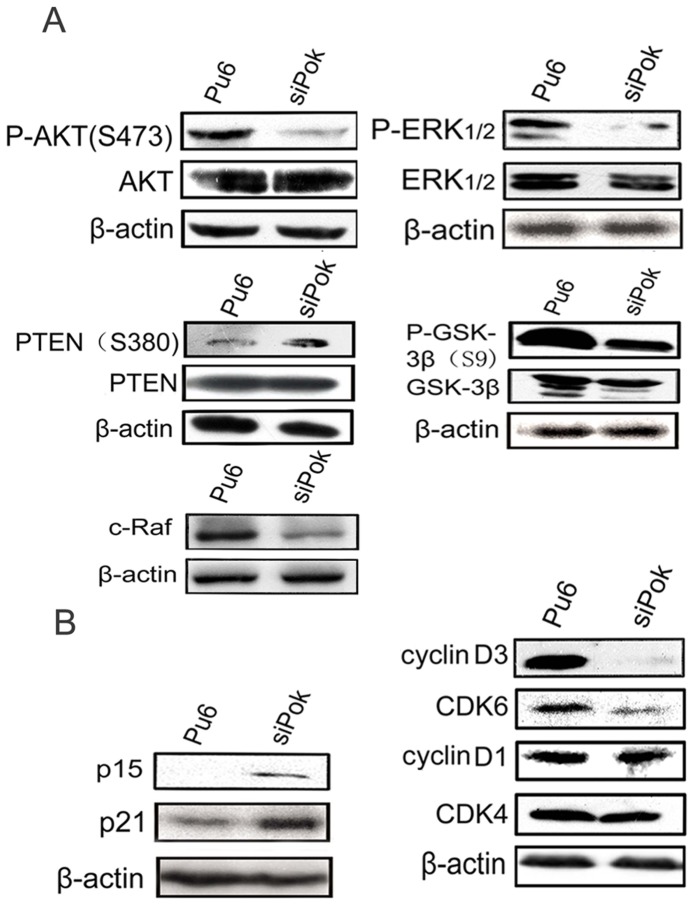
The effect of Pokemon on Akt and ERK signals as well as critical members of the cell cycle progression pathway. (**A**). Western blot analysis for the phosphorylation levels of Akt, Erk, PTEN, GSK-3β and c-Raf in HepG2-siPokemon and control cells. (**B**). Western blot analysis for cell cycle-related proteins in HepG2-siPokemon and control cells.

Previous studies have indicated that p14ARF, p53, NF-κB, Rb and p21 have roles in Pokemon-mediated carcinogenesis. Therefore, we aimed to determine whether Pokemon also regulates components of the (PI3K)/Akt and Raf/MEK/ERK pathways during the development of hepatocellular carcinoma. We used Western blot analysis to detect the phosphorylation levels of ERK and Akt, which mediate proliferation, survival, apoptosis and cell cycle progression. The expression levels of p-ERK1/2 and p-Akt (Ser473) were dramatically decreased following Pokemon knockdown in HepG2-siPokemon cells. In contrast, the expression of phospho-PTEN, which is a major negative regulator of the PI3/Akt pathway, was markedly increased in HepG2-siPokemon cells compared with control cells. We noted that the level of c-Raf, which indirectly activates ERK, was decreased in HepG2-siPokemon cells compared with control cells. We also assessed GSK-3β, which is a downstream target of the PI3 kinase/Akt pathway. As expected, the level of p-GSK-3β (S9), the inactivated form of the protein, was decreased in HepG2-siPokemon cells compared with control cells (Fig. 4A).

In addition to GSK-3β and the cyclin-dependent kinase inhibitor p21, we identified other effectors that regulate the cell cycle progression of HepG2 cells by analyzing the expression of several different cyclins, CDKs and CDKIs. We showed that the expression profiles of cyclin D3 and CDK6 were decreased in HepG2-siPokemon cells, whereas the expression levels of the cyclin-dependent kinase inhibitors p15 and p21 were upregulated. The expression levels of CDK4 and cyclin D1 were not significantly altered in HepG2-siPokemon cells compared with control cells (Fig. 4B). Based on these results, we confirmed that the knockdown of Pokemon in HepG2 cells suppressed the phosphorylation of ERK and Akt, thus activating GSK-3β, which is the downstream target of Akt. Silencing of Pokemon also suppressed the expression of cyclin D3 and CDK6 and upregulated the expression of p15 and p21.

### Pokemon knockdown suppressed the growth of xenografted tumors in nude mice

**Figure 5:**
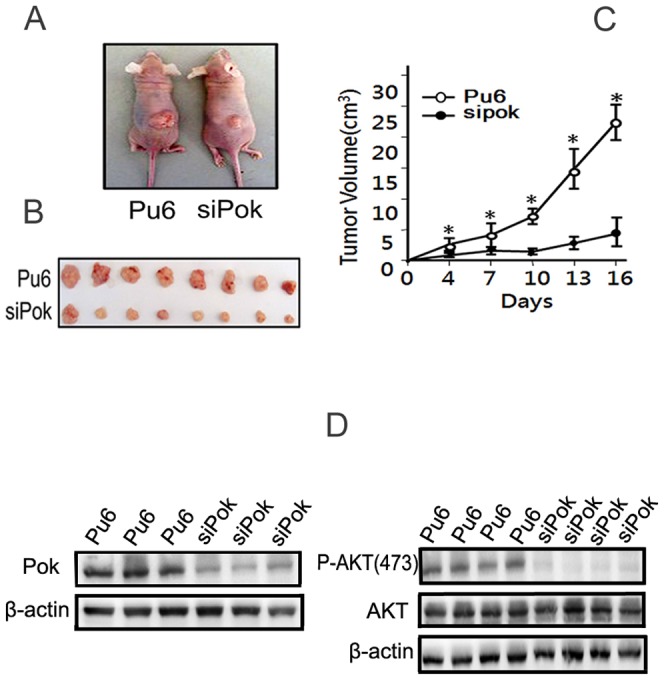
Pokemon silencing inhibits the proliferation of HepG2 cells in vivo. Nude mice were subcutaneously injected with HepG2-siPokemon or HepG2-Pu6 cells, and the tumors were excised after 16 days. (**A, B**). Representative images of tumors in nude mice. (**C**). The tumor volumes were recorded every three days for 16 days. The figure shows the mean ± SD (8 mice per group, *P<0.01). (**D**). Confirmation that Pokemon knockdown and decreased P-Akt levels are maintained in vivo.

We subcutaneously injected HepG2-siPokemon and HepG2-Pu6 cells in nude mice to induce xenograft tumors (Fig. 5A and B), and the tumors gradually enlarged over time (Fig. 5C). We measured the tumor size every 3 days. Our results indicated that the growth rates of tumors in nude mice that received transplants of HepG2-siPokemon cells were significantly lower than those in mice transplanted with HepG2-Pu6 cells (n = 8, *P<0.01). Western blot analysis of protein extracted from the tumor tissues showed that the Pokemon knockdown was maintained in the transplanted tumors in the nude mice, and the expression levels of p-Akt (Ser473) remained decreased in mice injected with HepG2-siPokemon cells compared with the control cells (Fig. 5D). Our results indicate that the absence of Pokemon was responsible for the inhibition of transplanted tumor growth in nude mice.

## Discussion

Currently, late diagnosis and high recurrence are the major causes of the poor overall survival of patients with HCC [22]. Therefore, it is necessary to elucidate the molecular mechanisms responsible for HCC progression and to identify efficient therapeutic targets. Previous studies have observed that Pokemon is overexpressed in HCC compared with benign or noncancerous tissue [5]. Previous reports indicated that the Pokemon expression level in HCC tissues is higher than that in adjacent noncancerous liver tissues. We confirmed this expression status and also verified the high expression of Pokemon in human HepG2 and Huh-7 cells. Then, we observed the functional changes in HepG2 and Huh-7 cells with stable Pokemon knockdown. The results of the MTT, BrdU, wound healing and transwell migration analyses demonstrated that the suppression of Pokemon expression in HepG2 and Huh-7 cells inhibited both cell proliferation and migration. We found that tumors in nude mice derived from transplanted HepG2-siPokemon cells displayed slower tumor growth than did tumors derived from HepG2-Pu6 cells. These results indicate that Pokemon mediates HCC development. However, the identification of the mechanisms involved requires further investigation.

Although the precise role of Pokemon in oncogenesis is unknown, previous studies have shown that Pokemon suppresses the transcription of target genes such as p14ARF, Rb and p21, which inhibits the expression of the anti-oncogenic gene p53 [7] and leads to cell cycle arrest. Interestingly, Pokemon enhances NF-κB-mediated transcription [8]. Our study investigated whether Pokemon targets any other signal transduction pathways in HCC cells to mediate changes in tumor cell proliferation and migration. Schmitz et al. [18] have reported that p-ERK1/2 and p-Akt(ser473) are both highly expressed in HCC tissues, and that the activation of the ERK and AKT pathways predicts poor prognosis in HCC. In addition, previous studies have reported Akt activation and impaired PTEN expression in 40% to 60% of human HCCs [2].

Chun-Ju Chang et al. [16] suggested that nuclear PTEN regulates cell proliferation and tumorigenesis through various signaling pathways, and that PTEN nuclear accumulation is regulated by S380 phosphorylation status. The increased nuclear localization of PTEN may protect the cells from DNA damage and tumorigenesis by modulating p53-dependent ROS reduction, cell cycle arrest, apoptosis, and possibly DNA damage repair. Moreover, nuclear P-PTEN can bind to p53 and enhance p53-mediated functions. Trotman et al. [23] showed that active nuclear PTEN can downregulate nuclear P-AKT, which was previously known to inactivate FOXO3a and accelerate tumor progression.

In this study, we confirmed that the activation of ERK and Akt was reduced in HepG2 cells in which Pokemon was knocked down. In addition, we showed that PTEN, which is a negative regulator of Akt, and c-Raf, which is an upstream regulator of MEK and ERK, were affected by Pokemon knockdown. We propose that Pokemon may regulate the PI3K/Akt and MEK/ERK pathways by affecting PTEN and c-Raf. Moreover, the inhibition of P-PTEN by Pokemon most likely downregulates p53-mediated functions, consequently promoting the progression of HCC. Nevertheless, the specific mechanisms involved require further investigation.

Previous studies have shown that the PI3K/Akt and MEK/ERK pathways mediate cell proliferation, migration and cell cycle progression [17], [24]. Akt regulates cell cycle progression by inducing GSK-3β inhibition [25], cyclin D1 degradation and p21 and p27 upregulation [26]. Pokemon has not been previously reported to impact any cell cycle regulator other than the CDKI p21^Waf1/Cip1^ [27]. Our FCM results did not show significant differences in cell cycle changes between HepG2-siPokemon cells and HepG2-Pu6 cells, but our Western blot analyses demonstrated that the knockdown of Pokemon induced the downregulation of cyclin D3/CDK6 complex formation and upregulated p15 and p21 expression and the activation of GSK-3β.

In summary, we conclude that Pokemon promotes the development of human hepatocellular carcinoma by regulating cell proliferation, migration and cell cycle progression, but no effect on cell apoptosis (Text S1 and Fig. S1). The underlying mechanism may involve the PI3K/Akt and c-Raf/MEK/ERK pathways. Pokemon also modulates the expression of cell cycle markers such as cyclin D3, CDK6, p15 and p21. Further investigation is required to determine whether cyclin D3, CDK6 and p15 are regulated by Akt- or ERK-dependent pathways.

To identify novel targets for the treatment of HCC, it is necessary to identify effective biomarkers and key proteins that mediate HCC development. Our study indicates that Pokemon plays an important role in the progression of HCC and may serve as a novel therapeutic target in a clinical setting.

## Supporting Information

Data S1 **Measurement of Caspase 3 and PARP activity.** The HepG2-siPok and HepG2-Pu6 cells were simultaneously treated with Cisplatin (8 ug/ml) for 24 h. Then, cells were harvested by centrifugation, and the pellets were washed twice in cold PBS. The cell pellets were lysed and the supernatants were used to determine the activities of caspase3 and PARP using the Caspase-3 antibody and PARP antibody (CST) in western blot analysis.
(DOC)

Figure S1 **The HepG2-siPok and HepG2-Pu6 cells were treated with Cisplatin, then the expression levels of Caspase 3 and PARP were determined using western blot analysis, and β-actin was used as a loading control (representative images from three of independent experiments).**
(TIF)
