# Supplementary material for: The Silencing of Pokemon Attenuates the Proliferation of Hepatocellular Carcinoma Cells In Vitro and In Vivo by Inhibiting the PI3K/Akt Pathway
Source: PLoS One. 2012 Dec 26;7(12):e51916. doi: 10.1371/journal.pone.0051916 (PMC3530584; doi:10.1371/journal.pone.0051916)
Supplement: Data S1 — Measurement of Caspase 3 and PARP activity. The HepG2-siPok and HepG2-Pu6 cells were simultaneously treated with Cisplatin (8 ug/ml) for 24 h. Then, cells were harvested by centrifugation, and the pellets were washed twice in cold PBS. The cell pellets were lysed and the supernatants were used to determine the activities of caspase3 and PARP using the Caspase-3 antibody and PARP antibody (CST) in western blot analysis. (DOC) [file pone.0051916.s001.doc]

**Supplementary data**

**Measurement of** **Caspase 3 and PARP activity**

The HepG2-siPok and HepG2-Pu6 cells were simultaneously treated with Cisplatin (8ug/ml) for 24 h. Then, cells were harvested by centrifugation, and the pellets were washed twice in cold PBS. The cell pellets were lysed and the supernatants were used to determine the activities of caspase3 and PARP using the Caspase-3 antibody and PARP antibody (CST) in western blot analysis.

**Figure Legend**

The HepG2-siPok and HepG2-Pu6 cells were treated with Cisplatin, then the expression levels of Caspase 3 and PARP were determined using western blot analysis, and β-actin was used as a loading control (representative images from three of independent experiments).

**Results**

**Knock down of Pokemon did not show obvious effects on HepG2 cell apoptosis**

To explore the role of Pokmeon on cell apoptosis, we used Cisplatin to induce cell apoptosis. The response to cell apoptosis was examined by caspase 3 and PARP activities. Caspases, a family of cysteine acid proteases, are central regulators of apoptosis. Apoptosis initiator caspases (including 8, 9, 10 and 12) are closely associated with proapoptotic signals. Once they activated, caspases cleave and activate downstream effector caspases (including 3, 6 and 7), which in turn cleave cytoskeletal and nuclear proteins like PARP, α-fodrin, DFF and lamin A to induce the apoptosis. Our analysis showed that both of Caspase 3 and PARP expression did not show significant differences in HepG2-siPok and HepG2-Pu6 cells. Thus, our data indicated that Pokemon do not play obvious function on Cisplatin induced HepG2 cell apoptosis.

**Discussion**

Our data showed that Pokemon regulates AKT- and ERK- pathways, which regulate cell proliferation, migration and apoptosis. In our analysis, Pokemon promotes cell proliferation and migration in HCC; however the cell apoptosis had not been significantly affected by silencing of Pokemon. We considered that apoptosis is a physiological process leading to cell death, and various regulators are involved in this complicated process. Silencing of Pokemon expression might be inhibit some apoptosis promoters and led to can not influence the overall apoptosis of HCC cells.
