# Supplementary figures and images for: The Silencing of Pokemon Attenuates the Proliferation of Hepatocellular Carcinoma Cells In Vitro and In Vivo by Inhibiting the PI3K/Akt Pathway
Source: PLoS One. 2012 Dec 26;7(12):e51916. doi: 10.1371/journal.pone.0051916 (PMC3530584; doi:10.1371/journal.pone.0051916)

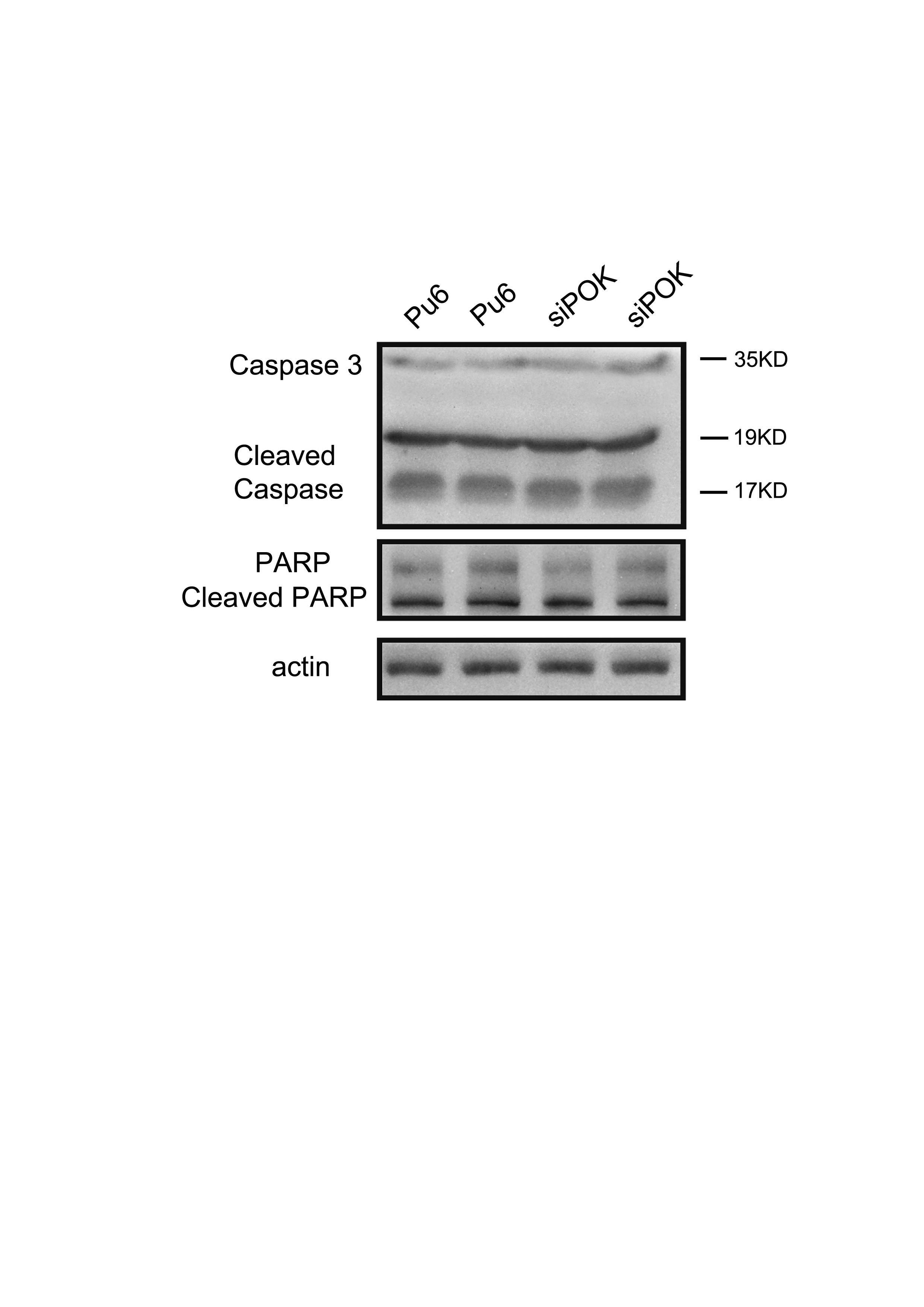

Supplement: Figure S1 — The HepG2-siPok and HepG2-Pu6 cells were treated with Cisplatin, then the expression levels of Caspase 3 and PARP were determined using western blot analysis, and β-actin was used as a loading control (representative images from three of independent experiments). (TIF) [file pone.0051916.s002.tif]
